# Nasal commensal *Staphylococcus epidermidis* enhances interferon-λ-dependent immunity against influenza virus

**DOI:** 10.1186/s40168-019-0691-9

**Published:** 2019-05-30

**Authors:** Hyun Jik Kim, Ara Jo, Yung Jin Jeon, Sujin An, Kang-Mu Lee, Sang Sun Yoon, Jae Young Choi

**Affiliations:** 1 Department of Otorhinolaryngology, Seoul National University College of Medicine, Seoul, Republic of Korea; 2 Seoul National University Hospital, Seoul, Republic of Korea; 3 Department of Microbiology and Immunology, Brain Korea 21 PLUS Project for Medical Sciences, Institute for Immunology and Immunological Diseases, Yonsei University College of Medicine, 50 Yonsei-ro, Seodaemun-gu, Seoul, 03722 Republic of Korea; 4 Department of Otorhinolaryngology, Yonsei University College of Medicine, 50 Yonsei-ro, Seodaemun-gu, Seoul, 03722 Republic of Korea; 5 Department of Otorhinolaryngology, Gyeongsang National University Hospital, Jinju, Republic of Korea

**Keywords:** Influenza A virus, Microbiome, Nasal commensal, Innate immunity, Interferon

## Abstract

**Background:**

*Staphylococcus epidermidis* is one of the most abundant colonizers of healthy human mucosa including that in the respiratory tract. As the respiratory microbiome has been linked to host immune responses, this study sought to determine the role of nasal mucosa-associated *S. epidermidis* in innate immune responses against the influenza A virus (IAV). *S. epidermidis* strains were isolated from nasal mucus samples of healthy individuals. The effects of these mucosa-derived commensal strains on interferon (IFN)-dependent innate immunity and IAV infection dynamics were tested in vitro using normal human nasal epithelial (NHNE) cells and human turbinate mucosa. The effects of *S. epidermidis* on antiviral immunity were also tested in vivo using an acute IAV infection mouse model.

**Results:**

Exposure of NHNE cells to nasal mucosa-derived *S. epidermidis* increased IFN-λ mRNA and secreted protein levels in the absence of viral stimulation. In the context of IAV infection, NHNE exposure to *S. epidermidis* prevented an increase in the viral burden, as revealed by IAV *PA* mRNA abundance, IAV nucleoprotein levels, and viral titers. *S. epidermidis* also enhanced transcription of IFN-stimulated genes independently of Toll-like receptor 2 and further induced IFN-λ production in IAV-infected cells by promoting phosphorylation of interferon regulatory factor 7. In a murine infection model, *S. epidermidis* prevented the spread of IAV to the lungs by stimulating IFN-λ innate immunity and suppressing IAV replication in the nasal mucosa.

**Conclusion:**

The human nasal commensal *S. epidermidis* mediates front-line antiviral protection against IAV infection through modulation of IFN-λ-dependent innate immune mechanisms in the nasal mucosa, thereby demonstrating the role of host-bacterial commensalism in shaping human antiviral responses.

**Electronic supplementary material:**

The online version of this article (10.1186/s40168-019-0691-9) contains supplementary material, which is available to authorized users.

## Background

The innate immune system of the respiratory epithelium serves as the first line of defense against respiratory viruses, including the influenza A virus (IAV), by producing interferon (IFN), a key molecule in the antiviral response [1]. IFNs including IFN-α, -β, -γ, and -λ enable substantial heterogeneity in host responses to respiratory viral infections, with specific IFNs interacting with different viruses to influence antiviral innate immune responses in the respiratory epithelium [1, 2]. Emerging evidence has indicated that among the IFN family of cytokines IFN-λ is a critical immune modulator against viral infection in the epithelial mucosa and the need for rapid immune responses to the respiratory virus is acquired by the activation of IFN-λ [3–7]. IFN-λ is believed to be primarily responsible for protection against viral invaders in the respiratory tract and to play an important role in local antiviral innate immunity [3, 5]. However, understanding of the modulators involved in IFN-λ production, especially within the context of in vivo respiratory viral infections, remains limited.

Human mucosal surfaces are in direct contact with the external environment and are, therefore, susceptible to invasion and colonization by various pathogens [8]. Studies on the clear reaction of the mucosal microbiome with the host increasingly take into consideration the contribution of mucosal immune responses and specific microbiome-mediated protection against infection from external pathogens to integrate environmental signals [9]. Respiratory mucosa, including that of nasal passages, is constantly exposed to inhaled pathogens, which directly impact the mucosal immune mechanisms [9, 10]. Inhaled pathogens encounter the host immune system for the first time in respiratory mucosa; especially, the nasal passage and microbial characteristics of the nasal mucus directly impact the mechanisms of initial immune responses [11–14]. Thus, insights into the microbiota of the human nasal mucosa can provide fundamental information regarding susceptibility to respiratory viral infections and factors contributing to related immune mechanisms, such as induction of IFNs [15, 16]. However, our knowledge of microbial composition in healthy nasal mucus is limited and the responses to inhaled pathogens or reasons for their colonization have not been comprehensively examined.

Based on increasing evidence of microbiome-regulated host immune homeostasis [11–13, 15], we assessed the microbial composition in healthy nasal mucus and subsequently investigated whether nasal commensal contributes to antiviral defense mechanisms in human nasal mucosa as a signaling modulator of antiviral immunity against IAV infection. The present study identified *Staphylococcus epidermidis* as its most abundant constituent and showed that *S. epidermidis* which were isolated from healthy human nasal mucus accelerated the clearance of IAV from nasal epithelium through IFN-λ-related immune responses. Furthermore, human nasal commensal *S. epidermidis* prevents IAV lung infection in mice by enhancing IFN-λ-related innate immune responses in the nasal mucosa. Overall, we present evidence of a key mechanistic link between the susceptibility to viral infections and nasal microbiome-mediated innate immunity.

## Methods

Additional methodological details are available in Additional file 1.

### Participant recruitment

Information on the 37 healthy subjects enrolled in this study and the exclusion criteria are described in Additional file 1. Participation was voluntary, with written informed consent obtained from all subjects. The Institutional Review Board of the Seoul National University College of Medicine approved the protocol for this study (IRB #C2012248 [943]).

### Sample collection

Mucus and/or nasal mucosa from the middle turbinates of the human subjects were collected and assessed for quality as described in Additional file 2: Movie S1).

### Nasal mucus microbiome characterization

For bacterial colony isolation, the mucus was placed in lysogeny broth (LB) plates. After 2 days incubation, bacterial colonies were obtained from the LB plates (Additional file 1: Figure S2) and the species of each colony were identified using GS-FLX 454 pyrosequencing by 16S rRNA gene amplification. *S. epidermidis* strains (N1-N4) from four individuals were used in the study (Additional file 1: Figure S2).

### Viruses and reagents

*Influenza A virus* strain A/Wilson-Smith/1933 H1N1 (IAV A/WS/33; ATCC, Manassas, VA, USA) was used in this study. Viruses were cultured and titrated using Madin-Darby canine kidney (MDCK) cells according to standard procedures [14].

### Cell culture and infection

Normal human nasal epithelial (NHNE) cells from five subjects were cultured using an air-liquid interface method [17]. Cells were used 14 days following the creation of the air-liquid interface. *S. epidermidis* and/or IAV infections are described in Additional file 1.

### Real-time PCR

Levels of transcripts encoding IFN-α, IFN-β, IFN-λ_1_, IFN-λ_2/3_, and IFN-γ, or IAV PA were determined using real-time PCR as described in Additional file 1.

### Quantification of secreted IFN-λ

Secreted human IFN-λ and mouse IFN-λ_2/3_ were quantified using Human IL-29/IL-28B (IFN-lambda 1/3) and Mouse IL-28A/B (IFN-lambda 2/3) DuoSet ELISA kits (R&D Systems, Minneapolis, MN USA), respectively. The working range of the assays was 62.5-4000 pg/ml.

### Viral titer determination

Viral titers were determined using a plaque assay as described in Additional file 1.

### Western blot analysis

IAV nucleoprotein (NP) levels and phosphorylation of IFN regulatory factor (IRF)3 and IRF7 were assessed using western blotting as described in Additional file 1.

### Murine infection model

Experiments with 7-week-old male C57BL/6J (B6) mice (Orientalbio, Seoul, Korea) were carried out according to guidelines approved by the Institutional Review Board of the Seoul National University College of Medicine (IACUC #2016-0093). Microbiome depletion, *S. epidermidis* and IAV infection, nasal lavage (NAL) and bronchoalveolar lavage (BAL) sample collection, and lung tissue harvesting are described in Additional file 1.

### Histological analysis

Fixing, hematoxylin/eosin staining, and histological analysis of the mouse lung tissues were carried out as described [18]. Peribronchiolar inflammation was scored using an 8-point scale. Lung sections from at least five mice, with at least six areas from each section, were examined. The five best sections were used for evaluation.

### Statistical analyses

The in vitro studies were performed at least in four independent cultures of NHNE cells from each donor. Differences between treatment groups were evaluated by analysis of variance (ANOVA) with a post hoc test. Between-group differences in studies in vivo, performed using five mice, were determined using Mann-Whitney *U* tests. Statistical analyses were performed using GraphPad Prism (v.5; GraphPad Software, La Jolla, CA, USA). A *p* value < 0.05 was considered significant.

## Results

### Characterization of bacterial communities in healthy nasal mucus

**Fig. 1:**
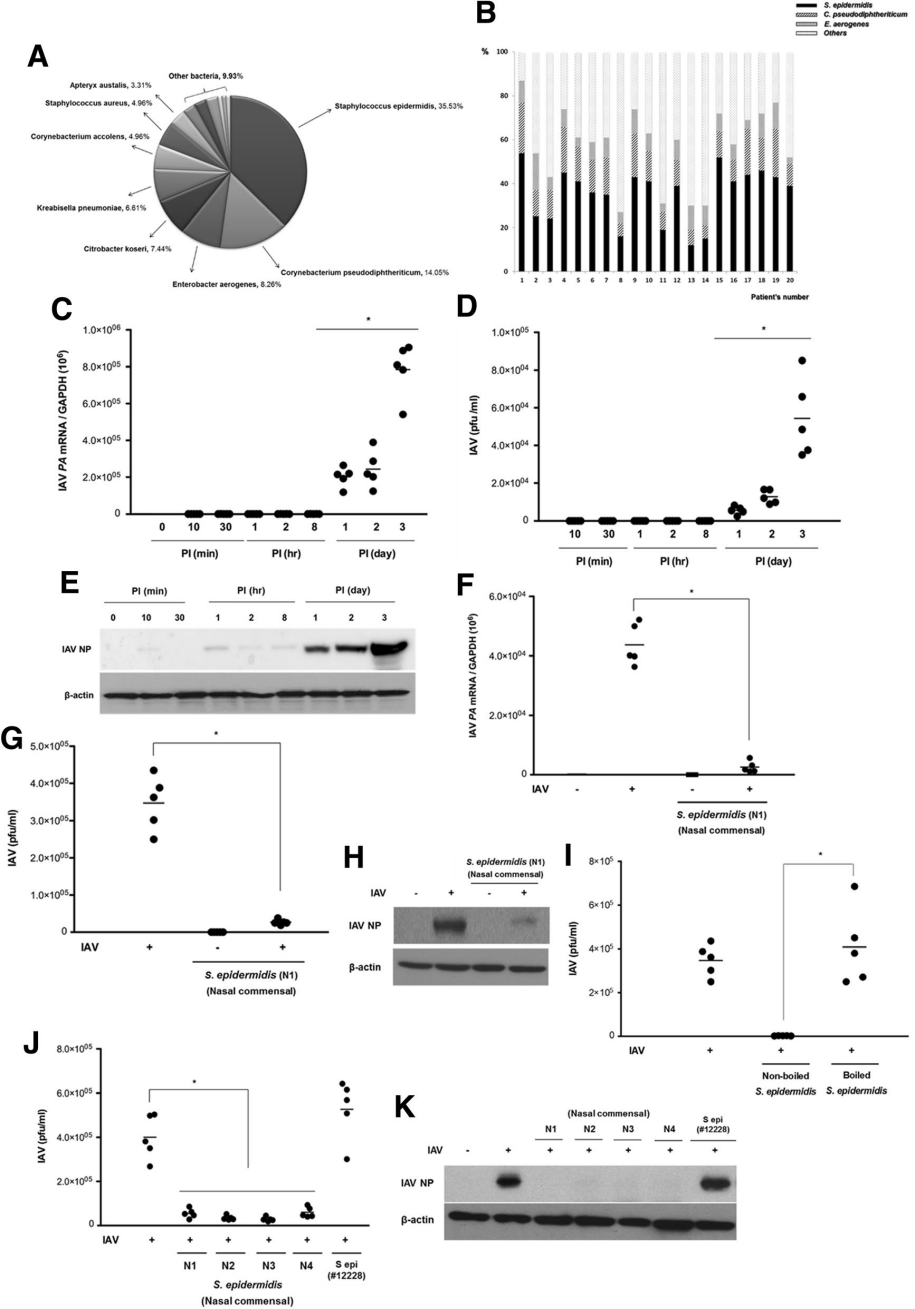
*S. epidermidis* exerts an antiviral effect against influenza A virus (IAV) infection in normal human nasal epithelial (NHNE) cells. **a** Bacterial species cultured from mucus samples obtained from the middle turbinates of healthy human subjects (*n* = 20) were identified via 16S rRNA gene sequencing. The distribution of the identified bacterial species is presented in the graph. **b** The relative abundance of nasal commensal, such as *S. epidermidis, C. pseudodiphtheriticum,* and *E. aerogenes*, in each subject. Bar graph presents the relative abundance of nasal commensal at the species level. NHNE cells from five healthy volunteers were inoculated with IAV at a multiplicity of infection (MOI) of 1. **c** IAV *PA* mRNA levels, normalized to cellular *GAPDH* transcript levels, were monitored by real-time PCR over the course of 3 days. **d** Viral titer of the infected NHNE cells was monitored using plaque assays. Results are presented as the mean ± standard deviation (SD) from five independent experiments. *p < 0.05 compared with mock-infected cells. **e** Levels of IAV nucleoprotein (NP) were monitored in NHNE cells following IAV infection using western blot analysis. Representative results are shown. PI post-infection. NHNE cells were inoculated with *S. epidermidis* N1 (MOI = 0.25) 8 h before IAV infection (MOI = 1). Subsequently, IAV *PA* mRNA levels (**f**), viral titers (**g**), and IAV NP protein levels (**h**) were assessed at 1 day post-infection (dpi) using real-time PCR, plaque assays, and western blotting, respectively. The mean viral titer was also measured in NHNE cells which were inoculated with boiled *S. epidermidis* and non-boiled *S. epidermidis* 8 h before IAV infection (**i**). Both mean viral titers (**j**) and IAV NP level (**k**) were determined at 1 dpi for NHNE cells infected with nasal mucus-derived *S. epidermidis* (N1, N2, N3, N4) and or non-pathogenic laboratory *S. epidermidis* ATCC 12228 prior to IAV infection. Real-time PCR and plaque assay results are presented as mean ± SD from five independent experiments. *p < 0.05 compared with the values for cells infected with IAV alone

The local microbiome of the middle turbinate mucus of healthy human subjects (*N* = 20) was analyzed by cultured bacterial colony and 16S rRNA gene sequencing. Based on at least 97% sequence identity, 46 bacterial species were detected in the middle turbinate mucus. *S. epidermidis*, *Corynebacterium pseudodiphtherticum, Enterobacter aerogenes, Citrobacter koseri, Klebsiella pneumoniae, Corynebacterium accolens, Staphylococcus aureus,* and *Apteryx australis* were among the most commonly identified species. *S. epidermidis* demonstrated the highest abundance, accounting for 35.53% of the mapped sequences (Fig. 1a). The abundance of nasal commensal *S epidermidis* was different in each patient, and the highest distribution reached 54.2%. The lowest abundance of *S. epidermidis* was 12.3% in the nasal commensal of healthy nasal mucus (Fig. 1b). To assess the role of this commensal organism in human nasal mucus, *S. epidermidis* strains isolated from four subjects (N1, N2, N3, and N4) were analyzed.

### *S. epidermidis* pretreatment suppresses IAV replication in the nasal epithelium

To analyze the effects of *S. epidermidis* pretreatment on the susceptibility of the nasal epithelium to IAV infection, NHNE cells from five healthy subjects were infected with IAV at a multiplicity of infection (MOI) of 1. Subsequently, IAV levels were assessed in the supernatants and cell lysates harvested at different time points post-infection. Real-time PCR revealed that IAV *PA* mRNA levels increased significantly starting at 1 day post-infection (dpi; mean IAV mRNA: 2.1 × 10^5^, 1 dpi; 2.4 × 10^5^, 2 dpi; 8.1 × 10^5^, 3 dpi; Fig. 1c). Plaque assays also found increased IAV titers starting at 1 dpi and a peak titer of 5.8 × 10^4^ plaque-forming units (PFU)/ml was observed at 3 dpi (Fig. 1d). Western blot analysis similarly revealed that IAV NP levels significantly increased by 1 dpi and continued to increase through to 3 dpi (Fig. 1e). These findings demonstrated the significant susceptibility of the nasal epithelium to IAV infection from 1 dpi.

To examine the effect of *S. epidermidis* on the antiviral immune response, NHNE cells were treated with *S. epidermidis* N1 (MOI = 0.25) at 8 h before inoculation of IAV (MOI = 1). The increase in IAV *PA* mRNA level (4.2 × 10^4^) in IAV-infected NHNE cells at 1 dpi was significantly attenuated by *S. epidermidis* (1.3 × 10^2^) (Fig. 1f). Viral titers were also significantly lower in the supernatants of NHNE cells co-infected with IAV and *S. epidermidis* than in those of cells infected with IAV alone (Fig. 1g). Similarly, *S. epidermidis* exposure also decreased IAV NP levels in IAV-infected NHNE cells (Fig. 1h). Interestingly, no reduction in viral load was observed in IAV-infected NHNE cells which were inoculated with boiled *S. epidermidis* (Fig. 1i). S*. epidermidis* strains isolated from the nasal mucus of other healthy subjects (N2, N3, and N4) also reduced IAV viral titer and protein levels (Fig. 1j, k). However, viral titers and NP levels were not reduced in NHNE cells infected with a non-pathogenic laboratory *S. epidermidis* ATCC 12228 strain [19] before IAV. These findings suggested that commensal *S. epidermidis* native to the human nasal passages can specifically suppress IAV replication in nasal epithelium.

### *S. epidermidis* preferentially induces IFN-λ in NHNE cells

**Fig. 2:**
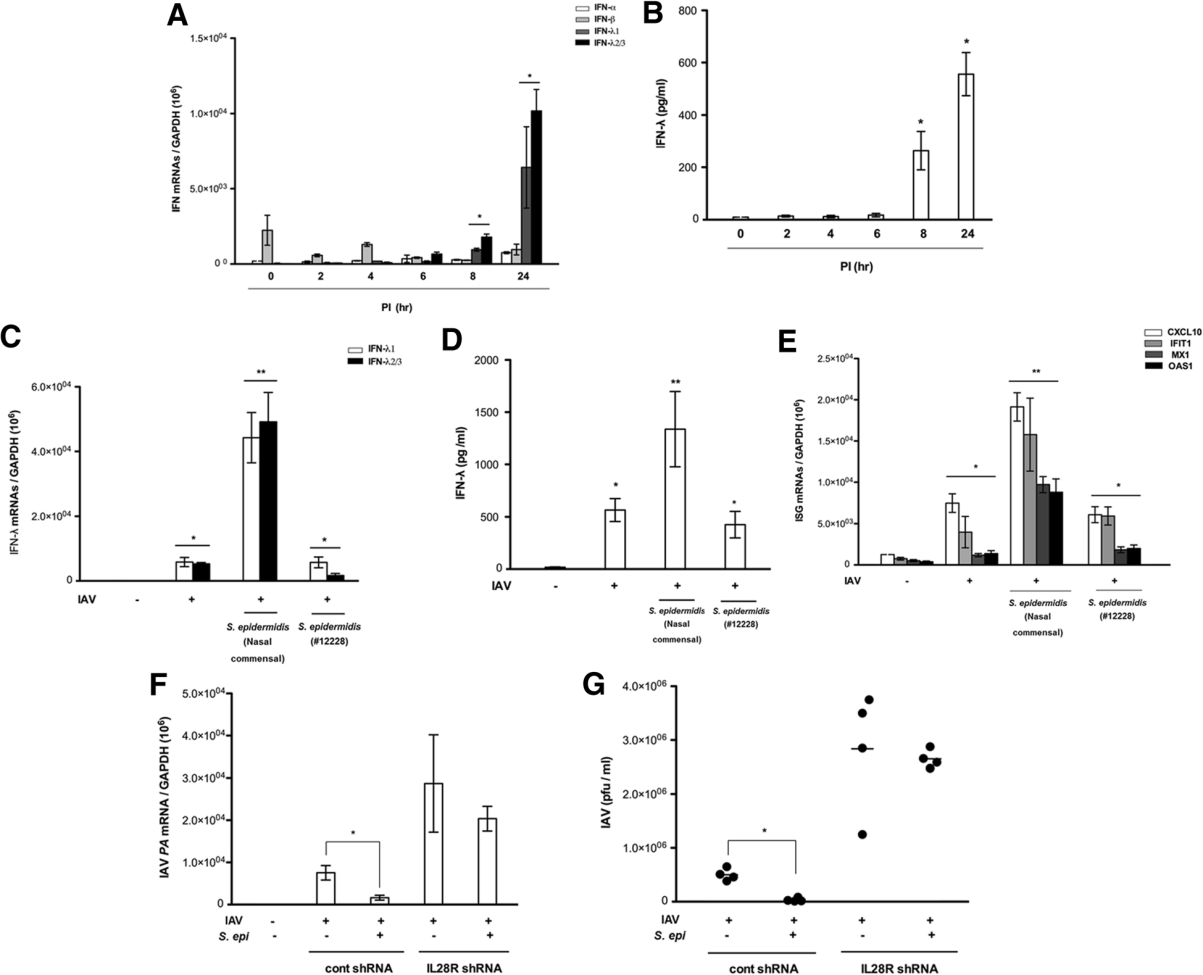
Influenza A virus (IAV) and *S. epidermidis* induce the expression of interferon (IFN) and IFN-stimulated genes (ISGs). Normal human nasal epithelial (NHNE) cells were inoculated with nasal mucus-derived *S. epidermidis* (N1, N2, N3, N4) at a multiplicity of infection (MOI) of 0.25. **a** Levels of mRNAs encoding IFNs were monitored by real-time PCR. **b** NHNE cell supernatants were assayed by ELISA for levels of secreted IFN-λ following *S. epidermidis* infection. IFN-λ mRNA abundance (**c**), IFN-λ protein levels (**d**), and ISG mRNA levels (**e**) were also assessed in NHNE cells infected with *S. epidermidis* and/or IAV. IAV *PA* mRNA levels (**f**) and viral titers (**g**) were also measured in NHNE cells which were transfected with control shRNA or IL28R shRNA. Results are presented as the mean ± SD from four independent *S epidermidis* experiments. *p < 0.05 compared with control NHNE cells. **p < 0.01 compared with NHNE cells infected with IAV alone

To assess the mechanisms of the *S. epidermidis-*dependent antiviral immune response in the nasal epithelium, we measured the expression of IFN genes in *S. epidermidis*-exposed NHNE cells. *S. epidermidis* isolated from healthy individuals (N1*,* N2, N3, and N4) significantly elevated the levels of *IFNL1* and *IFNL2/3* mRNA in NHNE cells starting at 8 h after treatment, with maximum levels observed 24 h after infection (*IFNL1* 6.4 × 10^3^, *IFNL2/3* 1.1 × 10^4^, Fig. 2a). *S. epidermidis* similarly induced the secretion of IFN-λ (1 dpi, 586.4 pg/ml, Fig. 2b) but did not increase the expression of genes encoding IFN-α, IFN-β, and IFN-γ.

When NHNE cells were inoculated with *S. epidermidis* prior to IAV, *S. epidermidis* induced IFN-λ more significantly in IAV-infected NHNE cells. NHNE cells treated with nasal commensal *S. epidermidis* 8 h before IAV infection demonstrated higher levels of gene expression (*IFNL1* 4.2 × 10^4^, *IFNL2/3* 4.8 × 10^4^) and secretion of IFN-λ (1412.4 pg/ml) than cells infected with IAV alone (*IFNL1* 5.6 × 10^3^, *IFNL2/3* 2.7 × 10^3^, 511.5 pg/ml) (Fig. 2c, d). However, pretreatment of NHNE cells with the non-pathogenic laboratory *S. epidermidis* ATCC 12228 strain before IAV infection did not result in a similar induction. Considering the more significant induction of IFN-λ observed, we subsequently assessed the effect of *S. epidermidis* on mRNA levels of the IFN-stimulated genes (ISGs) encoding CXCL10, IFIT1, Mx1, and OAS1, which are required for the innate immune response against IAV. Transcript levels of all four genes were significantly elevated in nasal commensal *S. epidermidis-*treated NHNE cells before IAV infection (CXCL10 1.9 × 10^4^, IFIT1 1.7 × 10^4^, Mx1 7.4 × 10^3^, OAS1 7.1 × 10^3^) (Fig. 2e) relative to untreated cells and those infected with IAV alone. In contrast, no induction was observed in cells pretreated with *S. epidermidis* ATCC 12228 prior to IAV infection.

As a next step, NHNE cells were transfected with short hairpin (sh)RNA of IL28R, which is a region of the IFN-λ receptor, to cause a functional loss of the IFN-λ-related signaling pathway. Interestingly, the significant decrease of IAV *PA* mRNA level (2.1 × 10^4^) and viral titer (1.1 × 10^6^ pfu/ml) in *S epidermidis*-treated NHNE cells before IAV infection was not observed in NHNE cells with transfection of *IL28R* shRNA (Fig. 2f, g). These data suggested that nasal commensal *S. epidermidis* induces IFN-λ production and secretion and facilitates IFN-related innate immune responses against IAV.

### *S. epidermidis* promotes IAV clearance independently of pattern recognition receptors

**Fig. 3:**
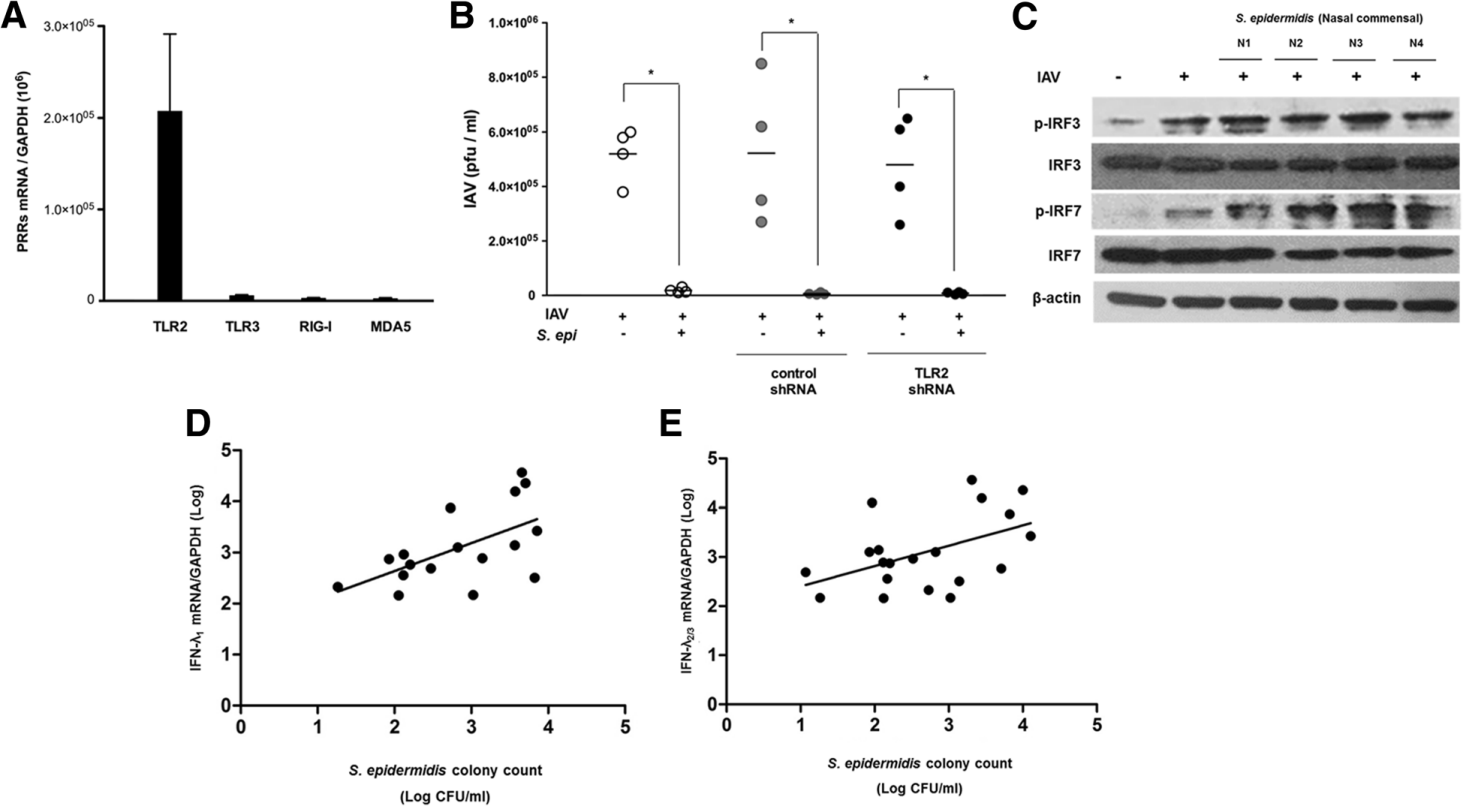
*S. epidermidis* induces interferon (IFN)-λ independently of pattern recognition receptors. **a** Abundance of transcripts encoding TLR2, TLR3, RIG-I, and MDA5 in normal human nasal epithelial (NHNE) cells infected with *S. epidermidis* 8 h before influenza A virus (IAV) infection was measured using real-time PCR at 1 day post-infection (dpi). **b** NHNE cells were transfected with control shRNA and *TLR2* shRNA, and plaque assays were performed to measure viral titers of IAV-infected NHNE cells in the presence or absence of *S. epidermidis*. Results are presented as the mean ± SD from four independent experiments. *p < 0.05 compared to control NHNE cells. **c** IAV- and *S. epidermidis*-induced phosphorylation of IFN regulatory factor (IRF) 3 and IRF7 was assessed using western blot analysis. The mRNA levels of IFN-λ_1_ (**d**) and IFN-λ_2/3_ (**e**) in the nasal mucosa from middle turbinate of healthy volunteers (*n* = 17), as measured by real-time PCR, were correlated with the colony-forming units (CFUs) of *S. epidermidis* isolated from the mucus of the middle turbinate collected from the same subjects. The correlation was determined by Spearman’s correlation analysis

To further investigate the mechanisms of significant IFN-λ induction by nasal mucus-derived *S. epidermidis*, we assessed mRNA levels of genes encoding pattern recognition receptors in NHNE cells infected with IAV in the absence or presence of *S. epidermidis* pretreatment. Transcripts encoding *TLR2, TLR3, RIG-I,* and *MDA5* were not more elevated in NHNE cells treated with *S. epidermidis* and IAV relative to those in cells infected with IAV alone (Additional file 1: Figure S1). The transcription levels of *TLR2* mRNA was higher in *S. epidermidis*-inoculated NHNE cells than *TLR3, RIG-I*, and *MDA5* at 1 dpi (Fig. 3a). Thus, we next assessed the impact of TLR2 on *S. epidermidis*-dependent modulation of IAV infection by transfecting NHNE cells with shRNA to suppress the expression of *TLR2* mRNA. Suppression of *TLR2* expression prior to IAV infection did not alter *S. epidermidis*-dependent effects on IAV PFU levels. Viral titers were reduced in NHNE cells treated with *S. epidermidis* before IAV infection (1.3 × 10^4^ pfu/ml) relative to those in cells infected with IAV alone in a manner independent of *TLR2* shRNA (4.3 × 10^5^ pfu/ml) (Fig. 3b).

To assess the *S. epidermidis*-related modulation of IFN-λ expression at the transcription level, we investigated the phosphorylation states of IFN regulatory factor (IRF) 3 and IRF7, which are required for transcription of IFN-λ in respiratory epithelium. Although total levels of IRF3 and IRF7 remained unchanged, those of the respective phosphorylated proteins were elevated in IAV-infected NHNE cells at 1 dpi relative to non-treated cells. Interestingly, levels of phosphorylated IRF7 were further increased in NHNE cells treated with *S. epidermidis* before IAV infection relative to those in cells infected with IAV alone (Fig. 3c). Together, these findings indicated that nasal commensal *S. epidermidis* can promote phosphorylation of IRF7 to induce IFN-λ expression and can suppress IAV replication independently of TLR2.

### *S. epidermidis* abundance and IFN-λ expression are positively correlated in human nasal mucosa

Considering the in vitro effect of nasal commensal *S. epidermidis* strains on IFN expression and IAV infection, we next investigated the relationship between *S. epidermidis* abundance and IFN-λ mRNA levels. Nasal mucus and middle turbinate mucosa of 17 healthy subjects were collected and assessed for the number of *S. epidermidis* colony-forming units (CFU) and IFN-λ_1_ and IFN-λ_2/3_ mRNA levels were measured using turbinate mucosa. Nasal commensal *S. epidermidis* CFUs positively correlated with mRNA levels of IFN-λ_1_ (*r* = 0.566, *p* = 0.018) and IFN-λ_2/3_ (*r* = 0.446, *p* = 0.049) in the nasal mucosa of the same subjects (Fig. 3d, e). These data indicated that the levels of *S. epidermidis* in the nasal mucus proportionally affect the transient expression of IFN-λ in nasal mucosa.

### *S. epidermidis* pretreatment prevents IAV lung infection in vivo through suppression of IAV replication in the nasal mucosa.

**Fig. 4:**
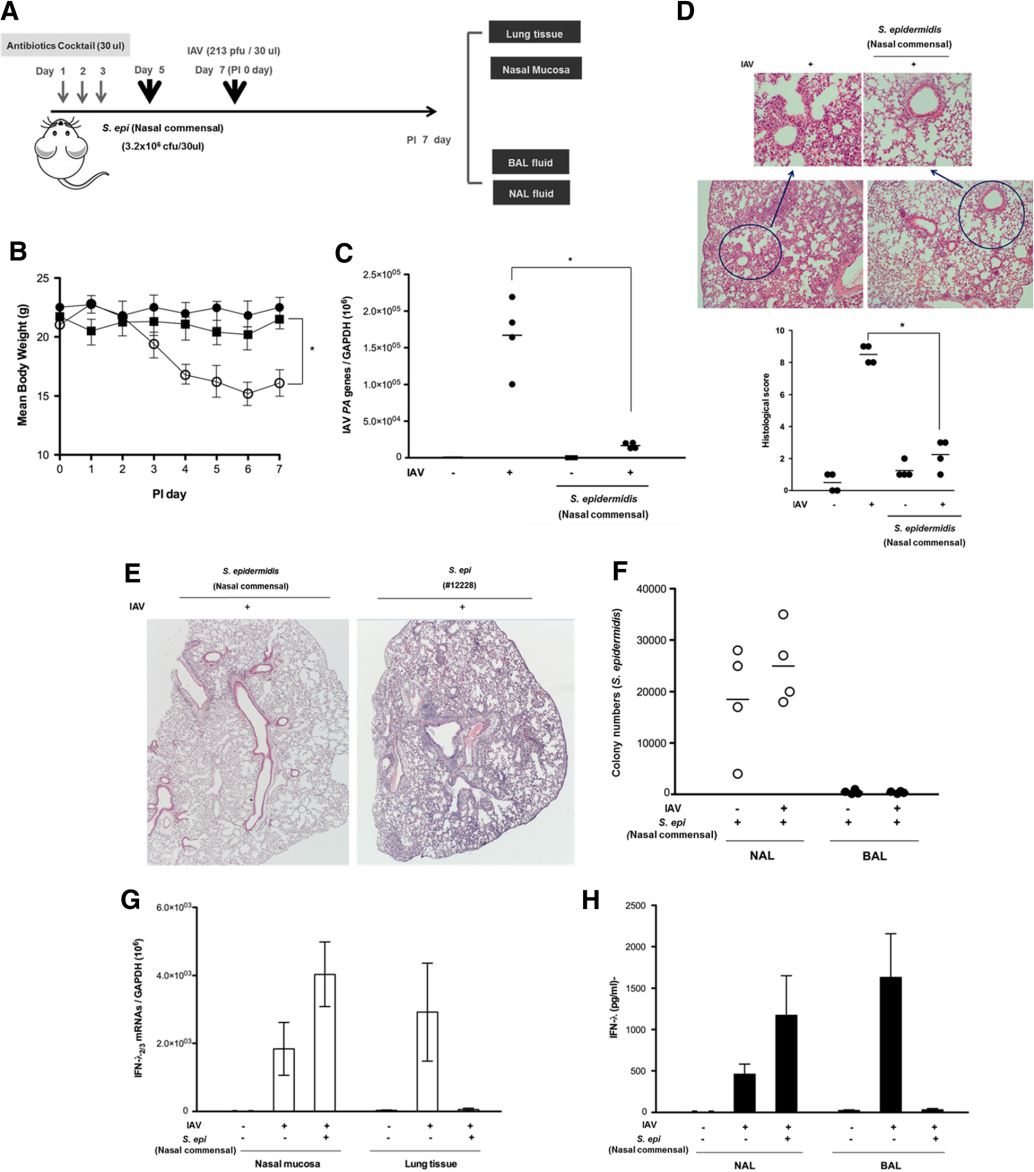
Human nasal mucus-derived *S. epidermidis* induces interferon (IFN)-λ production and suppresses infection spread in influenza A virus (IAV)-infected mice. **a** Schematic of the mouse model experimental design. The native microbiome of the C57BL/6J mice was depleted with an antibiotic regimen prior to inoculation. The mice (*N* = 4) were inoculated with *S. epidermidis* (3.2 × 10^6^ CFU/30 μl PBS) and/or with IAV (213 PFU) at the indicated time points. **b** Mean body weight of *S epidermidis*-inoculated mice with or without IAV infection was measured. IAV *PA* mRNA levels in the mouse lung tissue (**c**) were assessed at 7 days post-infection (dpi) and hematoxylin and eosin (H&E)-stained micrographs (**d**) were also generated from lung sections obtained at 7 dpi. B6 mice were inoculated with human nasal mucus-derived *S. epidermidis* and non-pathogenic laboratory *S. epidermidis* 12228 before IAV infection and H&E-stained micrographs (**e**) were also generated from lung sections obtained at 7 dpi. Micrographs shown are representative of lung sections from four mice. The micrographs were used to assess inflammation and tissue damage and to calculate a histological score. **f** *S. epidermidis* CFUs in the NAL and BAL fluid samples were determined at 7 dpi. **g** IFN-λ_2/3_ mRNA levels in mouse nasal mucosa and lung tissue at 7 dpi were measured using real-time PCR. **h** Concentrations of mouse NAL and BAL fluid samples were compared to secreted IFN-λ concentrations using ELISA. Real-time PCR, plaque assay, and ELISA results are presented as mean ± SD from four independent experiments. *p < 0.05 compared with mice infected with IAV alone

Considering our observations, we next assessed the *S. epidermidis* anti-IAV protective properties in vivo using a murine model of infection. The nasal cavities of B6 mice (*N* = 4) were inoculated with human nasal mucus-derived *S. epidermidis* at 2 days (day 5) following nasal microbiota-depletion (days 1, 2, and 3) using 30 μl of an antibiotic cocktail (vancomycin, neomycin, ampicillin, and metronidazole). Then, *S. epidermidis*-inoculated B6 mice were infected with IAV (213 pfu/30 μl) at 2 days after *S. epidermidis* inoculation (day 7) (Fig. 4a).

As gross determinants of virus-induced morbidity, the body weights and survival rates of the infected B6 mice were monitored for 7 days. IAV-infected mice exhibited a significant decrease in mean body weight with an 80% survival rate until 7 dpi. Interestingly, B6 mice that were administered human nasal mucus-derived *S. epidermidis* before IAV infection did not exhibit a significant weight loss until 7 dpi and the mean body weight of these mice exceeded 20 g until 7 dpi resulting in 100% survival of mice after IAV infection (Fig. 4b).

Compared to mice infected with IAV alone (2.8 × 10^5^), those infected with IAV following *S. epidermidis* inoculation showed lower IAV *PA* mRNA levels (4.2 × 10^4^) in the lung tissue (Fig. 4c) and the *S. epidermidis*/IAV exposure also resulted in attenuated pathologic findings in the lung, with significantly lower histologic scores (Fig. 4d). However, significant lung pathologic findings were obtained from B6 mice that were administered the non-pathogenic laboratory *S. epidermidis* ATCC 12228 strain before IAV infection compared to the mice that were inoculated with human nasal mucus-derived *S. epidermidis* (Fig. 4e).

We next assessed the distribution of bacteria in *S. epidermidis*-inoculated mice by comparing the CFUs in the NAL and BAL samples. Whereas substantial numbers of *S. epidermidis* CFUs were observed in the NAL samples, the levels of *S. epidermidis* cells in the BAL samples were under the detection limit (Fig. 4f). We also asked whether these differences in *S. epidermidis* distribution translate into specific patterns of IFN-λ expression in the mice. IAV infection alone resulted in elevated *IFNL2/3* gene expression in the nasal mucosa and lung tissue, as well as in increased secreted IFN-λ levels in NAL and BAL samples. Gene expression induction and IFN secretion were further enhanced in the nasal mucosa and the NAL fluid, respectively, of IAV-infected mice pre-inoculated with *S. epidermidis*. However, IFN-λ_2/3_ mRNA and IFN-λ protein levels were not detectable in the lung tissue and BAL fluid of these pre-inoculated animals accompanied with completely lower viral replication in vivo lung (Fig. 4g, h). Together, these findings demonstrated that the human nasal commensal *S. epidermidis* can boost the IFN-λ-dependent innate immune response in mouse nasal mucosa, thereby suppressing IAV replication at the level of the nose and preventing acute IAV lung infection.

## Discussion

Our study revealed that *S. epidermidis* is the most abundant microbiome that colonizes healthy human nasal mucus and the distribution of *S. epidermidis* might be significantly associated with IFN-λ-dependent innate immune responses in the nasal mucosa. Our findings also imply that the intranasal administration of *S. epidermidis* is a potential strategy for the prevention of respiratory viral infections by controlling viral replication at the level of the nasal mucosa. This study presents novel evidence of the role played by the healthy human nasal microbiome in the defense against IAV replication in the nasal mucosa.

Respiratory mucosa is the first target organ for environmental pathogens, including respiratory viruses. Recent work has highlighted the critical role of respiratory mucosa as a barrier for restricting the invasion of the host by multiple pathogens [10, 20–22]. The compositional and predicted functional differences in the respiratory microbiome resulting from environmental stimuli have been gaining increasing interest and the importance of the respiratory microbiome, especially with respect to immune protection, has been greatly recognized [23–28]. Therefore, understanding the composition and potential adverse changes in the respiratory mucosal microbiome is essential for developing new approaches for the prevention and treatment of respiratory infections. The nasal mucosa is also a key player in immunological defense to protect the respiratory tract and is responsible for filtration of inhaled pathogens from direct exposure to pressurized airflow [21, 22]. There is also growing evidence that a microbiome community resides in the nasal mucus, which is secreted by epithelial cells, and covers the whole surface of the mucosa, and we found that *S. epidermidis* is the most abundant commensal organism in the human nasal mucus, accounting for ~ 35% of the identified bacterial species. Human respiratory viruses first encounter host defense mechanisms in the upper or lower respiratory epithelium [29–34]. Host protection against viral infections can be conferred by innate resistance, with a specialized innate immune system of nasal epithelium capable of combating invasion by respiratory viruses [10]. Thus, we focused on the contribution of *S. epidermidis* to antiviral innate immune defense mechanisms of the nasal mucosa.

Antiviral innate immunity in the respiratory tract is mediated by an increase in IFN secretion [35–41]. The role of type 1 IFNs in antiviral innate immune responses and the activation of the adaptive immune system have been well documented [42–44]. Increasing evidence shows that IFN-λ is also critical for antiviral innate immunity in the respiratory tract, with disrupted IFN-λ-related innate immunity increasing susceptibility to respiratory viral infections [45–48]. Some studies have reported that IFN-λ is the primary cytokine that mediates the antiviral response against rhinovirus and influenza virus in the lungs [46, 49–51]. We have also reported that IFN-λ represents the predominant IFN type induced by IAV and contributes to the first-line defense against viral infections in human nasal epithelial cells [10]. We have concentrated on verifying the immune mediators for IFN-λ in the nasal mucosa and propose that IFN-λ can be regulated by the nasal microbiome, with *S. epidermidis* as the most abundant commensal organism. Here, we found that nasal commensal *S. epidermidis* showed potent antiviral activity in nasal epithelium and all ensuing antiviral responses from *S. epidermidis* are dependent on the production of IFN-λ without affecting IFN-α, IFN-β, and IFN-γ expression because responses are lost in nasal epithelial cells lacking specific receptors for IFN-λ, as shown in Fig. 2f and g. This hypothesis is underscored by the positive correlation between IFN-λ mRNA levels of nasal mucosa and *S. epidermidis* colony numbers in human nasal mucus. Thus, a higher abundance of *S. epidermidis* in the nasal mucus of healthy individuals results in higher baseline IFN-λ levels in the absence of a viral infection.

We furthermore demonstrated that the nasal commensal *S. epidermidis* confers protection against IAV infection. Relative to infection with IAV alone, inoculation of *S. epidermidis* before IAV infection reduced the viral burden of NHNE cells, while concomitantly inducing IFN-λ and ISG expression. Previous research has shown that *S. epidermidis* directly binds influenza virus, with the extracellular matrix-binding protein Embp identified as a major contributor to the anti-influenza effect of *S. epidermidis* ATCC 12228 [19]. However, in our in vitro system using full differentiated human nasal epithelium, the laboratory *S. epidermidis* ATCC 12228 strain was not capable of reducing the viral burden of IAV-infected NHNE cells or of inducing IFN-λ production. We speculate that the nasal commensal *S. epidermidis* possess specific characteristics, distinct from those of non-pathogenic laboratory *S. epidermidis* ATCC 12228 or of respiratory tract pathogens, that allow it to boost baseline immune mechanisms through IFN-λ. These findings suggest that commensal *S. epidermidis* specifically from human nasal mucus shows distinctive antiviral immune responses against IAV through induction of IFN-λ from at least 8 h after inoculation.

In addition to showing in vitro activity in NHNE cells, nasal mucus-derived *S. epidermidis* also suppressed IAV-caused lung infection in vivo. Inoculation of mice with human commensal *S. epidermidis* strains prior to IAV infection increased the expression of IFN-λ in the mouse nasal mucosa and prevented IAV-induced lung tissue damage. We hypothesize that the mice that received the human commensal bacteria prior to IAV infection had limited IAV replication in their nasal mucosa, thereby preventing acute lung infection. We did not observe direct binding between human commensal *S. epidermidis* and IAV in vitro or in vivo.

The current findings indicate that nasal commensal *S. epidermidis* could be detected by TLR2 in the nasal epithelium but *S. epidermidis* induced IFN-λ through direct signal transduction to transcription factors regardless of recognition by TLR2. Both IRF3 and IRF7 have been characterized as transcription factors for IFNs and IFN-λ induction depends on direct signal transduction through the phosphorylation of IRF3 and IRF7 [48, 52]. While IRF3 is generally regarded as the transcription factor required for the initiation of IFN transcription, IRF3 deficiency has little impact on IFN expression [52]. In contrast, lack of IRF7 reduced IFN production significantly in airway epithelium [52]. Our data explain how nasal commensal *S. epidermidis* could promote phosphorylation of IRF7 rather than IRF3 and finally induce IFN-λ-related immune responses in nasal epithelium. We did not show the definite paradigm for *S. epidermidis*-enhanced phosphorylation of IRF7 in nasal epithelium. We just speculate that *S. epidermidis*-secreted molecules interact with IRF7 and are primarily responsible for the phosphorylation of IRF7 for the more potent induction of IFN-related antiviral immune responses in airway epithelium.

As shown in Fig. 1i and j, it is of interest that the non-pathogenic *S. epidermidis* strain (ATCC #12228) failed to induce protective effects against IAV infection. This supports the notion that the *S. epidermidis* strains recovered from the human nasal cavity might have acquired novel genetic features during the long-term symbiosis in the human nasal mucosa. The comparative genomic analysis will provide insights into the genetic determinants that help us understand mechanisms by which our innate immunity is activated in response to the symbiotic interaction with a commensal microbe.

## Conclusion

Our study provides a greater understanding of how the nasal microbiome enhances IFN-dependent innate immune responses to protect the respiratory tract against influenza viral infection. We showed that the abundant human nasal commensal organism *S. epidermidis* enhances resistance against IAV infection in human nasal epithelium through IRF7-dependent IFN-λ amplification and prevents IAV lung infection through the suppression of IAV replication at the level of the nasal mucosa. Thus, intranasal delivery of the human nasal commensal *S. epidermidis* represents a potential therapeutic approach for preventing and treating respiratory viral infections via induction of IFN-λ-related innate immune responses.

## Additional files

Additional file 1: Supplementary material and methods with supplementary figures. (DOC 1276 kb)

Additional file 2: **Movie S1.** Mucus and/or nasal mucosa from the middle turbinates of the human subjects. (AVI 4230 kb)

## Data Availability

Not applicable

## Abbreviations

B6: C57BL/6
BAL: Bronchoalveolar lavage
CFU: Colony-forming unit
DPI: Day post-infection
IAV: Influenza A virus
IFN: Interferon
IRF: Interferon regulatory factor
ISG: Interferon-stimulated genes
MDCK: Madin-Darby canine kidney
MOI: Multiplicity of infection
NAL: Nasal lavage
NHNE: Normal human nasal epithelial
NP: Nucleoprotein
TLR: Toll-like receptor
