# Supplementary material for: Nasal commensal Staphylococcus epidermidis enhances interferon-λ-dependent immunity against influenza virus
Source: Microbiome. 2019 May 30;7:80. doi: 10.1186/s40168-019-0691-9 (PMC6542144; doi:10.1186/s40168-019-0691-9)

**Supplementary Material and Methods**

**Participant Recruitment**

A total of thirty seven healthy subjects were enrolled in this study: 21 males (mean body mass index 21.5 kg/m^2^) and 16 females (mean body mass index 22.6 kg/m^2^), whose mean age was 35.2 years, and who were referred to the Department of Otorhinolaryngology Seoul National University Hospital (Seoul, Korea) primarily for septal surgery between March 2016 and Jan 2017. Intranasal endoscopy, computed tomography of the paranasal sinus, and a skin allergy test were performed before sampling; none of the subjects showed signs of infection, and all showed negative results in the allergy test. Those subjects who had taken any kinds of antibiotics within the prior 2 months; who were pregnant or smokers; who had diseases and medication histories related to asthma; and who had any other chronic diseases such as atherosclerosis, hypertension, arrhythmia, congestive heart failure, diabetes mellitus, osteoporosis, hepatitis, cancer, and autoimmune or neurological diseases were excluded. Participation was voluntary, and written informed consent was obtained from all participants and the Institutional Review Board of the Seoul National University College of Medicine approved the protocol of this study (IRB number C2012248 [943]).

**Sample collection**

The mucus from the middle turbinate was collected individually using sterile 3M Quick swabs (3M Microbiology Products, ST Paul, MN, USA) from 20 subjects using a rigid 0-degree endoscope in an operating room (Supplementary video file, left nasal cavity). The swabs were inserted into the nasal cavities of the subjects without touching either the nostril or the anterior part of inferior turbinate, which are lined with stratified squamous epithelium, not respiratory epithelium. The swabs were then gently rotated around the middle turbinate. The swabs with mucus were fixed in a fixative solution and were transported immediately to the laboratory for identification and subsequent microbial analysis. For bacterial colony isolation, the mucus was placed in Lysogeny broth (LB) plates. After 2 day incubations, bacterial colonies were obtained from LB plates (Fig. S1) and the species of each colony were identified using GS-FLX 454 Pyrosequencing by 16S rRNA Gene Amplification.

To detect possible contamination, negative controls were prepared and subjected to the same procedures. The first step in the analysis was extraction of DNA from the bacterial pellet; the bacterial DNA from nasal or oropharyngeal mucosal swab samples was extracted with the Fast DNA Spin Kit for Soil (MP Biomedicals, Solon, Ohio, USA). The extracted DNA was dissolved in sterile water containing 40 μg/mL RNase A and was quantified with a Nano Quant Infinite M200 spectrophotometer (Tecan, Männedorf, Switzerland) by the ratio of absorbance values at 260 and 280 nm (A_260_/A_280_). Nasal mucus from 48 subjects that showed stable and significant genomic DNA levels were included in the present study.

**Viruses and reagents**

Influenza A virus (IAV WS/33: H1N1, ATCC, Manassas, VA, USA) was used to induce acute viral lung infection. Virus stocks were grown in Madin-Darby canine kidney cells in virus growth medium according to a standard procedure [14]. Briefly, after 48 h incubation at 37°C, the supernatants were harvested and spun by centrifugation at 5000 rpm for 30 min to remove cellular debris. Virus stocks were titrated on MDCK cells using a tissue culture infectious dose assay and stored at -80°C.

**Cell culture**

Normal human nasal epithelial (NHNE) cells were cultured as described previously [17]. Brieﬂy, passage-2 NHNE cells (1 x 10^5^ cells/culture) were seeded in 0.25 ml of culture medium on Transwell clear culture inserts (24.5-mm, with a 0.45-mm pore size; Costar Co., Cambridge, MA, USA). Cells were cultured in a 1:1 mixture of basal epithelial growth medium and DMEM containing previously described supplements. Cultures were grown while submerged for the ﬁrst 9 days. The culture medium was changed on Day 1, and every other day thereafter. An air–liquid interface (ALI) was created on Day 9 by removing the apical medium and feeding the cultures from the basal compartment only. The culture medium was changed daily after the initiation of the ALI. We add antibiotics such as 1 % penicillin and streptomycin into the all media for subculture and culture stages and we also add antifungal agent, fungizone (1 ml / 1000 ml media) (Life technologies, Grand island, NY, USA) after filtering the media. All experiments described here used cultured nasal epithelial cells at 14 days after the creation of the ALI.

**Real-time PCR**

NHNE cells were infected with WS/33 (H1N1) for 10, 30 min, 1, 2, 8 hr, 1, 2, 3 days and total RNA was isolated using TRIzol (Life technology, Seoul, Korea). cDNA was synthesized from 3 μg of RNA with random hexamer primers and Moloney murine leukemia virus reverse transcriptase (Perkin Elmer Life Sciences, Waltham, MA, USA and Roche Applied Science, Indianapolis, IN, USA). Amplification was performed using the TaqMan Universal PCR Master Mix (PE Biosystems, Foster City, CA, USA) according to the manufacturer’s protocol. Briefly, amplification reactions had a total volume of 12 μl and contained 2 μl of cDNA (reverse transcription mixture), oligonucleotide primers (final concentration of 800 nM), and TaqMan hybridization probe (200 nM). Real-time PCR probes were labeled at the 5’ end with carboxyfluorescein (FAM) and at the 3’ end with the quencher carboxytetramethylrhodamine (TAMRA). To quantify the cellular viral level and host gene expression, cellular RNA was used to generate cDNA. The IAV level was monitored using a quantitative PCR for the *PA* gene (segment 3) with forward and reverse primers and probe 5’-ggccgactacactctcgatga-3’, 5’-tgtcttatggtgaatagcctggttt-3’, and 5’-agcagggctaggatc-3’, respectively. Primers for Human or mouse IFN-α, IFN-β, IFN-λ_1_, IFN-λ_2/3_ and IFN-γ were purchased from Applied Biosystems (Foster City, CA, USA). Real-time PCR was performed using the PE Biosystems ABI PRISM® 7700 Sequence Detection System. Thermocyling parameters were as follows: 50°C for 2 min, 95°C for 10 min, and then 40 cycles of 95°C for 15 s and 60°C for 1 min. Target mRNA levels were quantified using target-specific primer and probe sets for IAV WS/33 (H1N1), IFN-α, IFN-β, IFN-λ_1_, IFN-λ_2/3_ and IFN-γ. All PCR assays were quantitative and utilized plasmids containing the target gene sequences as standards. All reactions were performed in triplicate, and all real-time PCR data were normalized to the level of the housekeeping gene glyceraldehyde phosphate dehydrogenase (GAPDH, 1×10^6^ copies) to correct for variations between samples.

**Quantification of secreted IFN-λ**

Secreted human IFN-λ (DY1598B) and mouse IFN-λ_2/3_ (DY1789B) were quantified using a Duoset ELISA kit from R&D Systems according to the manufacturer’s instructions for NAL fluid. The working range of the assay was 62.5-4000 pg/ml.

**Plaque assay**

Virus samples were serially diluted with PBS. Confluent monolayers of MDCK cells in six-well plates were washed twice with PBS and then infected in duplicate with 250 μl/well of each virus dilution. The plates were incubated at 37°C for 45 min to facilitate virus adsorption. Following adsorption, a 1% agarose overlay in complete MEM supplemented with TPCK trypsin (1 μg/ml) and 1% fetal bovine serum was applied. The plates were incubated at 37°C, and cells were fixed with 10% formalin at two days post of infection.

**Mice and virus inoculation**

Male C57BL/6J (B6) mice (Orientalbio, Seoul, Korea) aged 7 weeks (19–23 g) were used as wild-type (WT) mice. B6 mice used in the study, like other commercially-available strains of inbred mice, carry a dysfunction Mx1 gene and are not congenic B6 mice with a function Mx1 gene, derived from influenza-resistant mice. All experiments were approved by the Institutional Review Board of the Seoul National University College of Medicine (IACUC number 2016-0093) and the research methods were carried out in accordance with the approved guidelines.

For infections, *S epi* (3.2x10^6^ cfu in 30 ul PBS) and IAV (WS/33, H1N1; 213 pfu in 30 μl PBS) were inoculated into WT mice by intranasal delivery. After euthanizing the mice, bronchoalveolar lavage (BAL) fluid was obtained from the lungs by lavaging with 1000 μl 0.5 mM ethylene diamine tetraacetic acid (EDTA) in phosphate-buffered saline (PBS) after cannulation of the trachea. The BAL fluid was used for enzyme-linked immunosorbent assay (ELISA) for measuring secreted protein levels and plaque assay to determine the viral titer. Mouse lung tissue was also harvested for real-time polymerase chain reaction (PCR), microarray, and immunohistochemistry analyses.

**Immunohistochemistry and histologic analysis**

Lung tissue was fixed in 10% (vol/vol) neutral buffered formalin and embedded in paraffin. Paraffin-embedded tissue slices were stained with hematoxylin/eosin (H&E) or periodic acid–Schiff (PAS) solution (Sigma, Deisenhofen, Germany). Histopathologic analysis of inflammatory cells in H&E-stained lung sections was performed in a blinded fashion using a semi-quantitative scoring system as previously described [18]. Lung sections from at least five mice were examined. Briefly, peribronchiolar inflammation were scored as follows: 0, normal; 1, a few cells; 2, a ring of inflammatory cells one layer deep; 3, a ring of inflammatory cells two to four cells deep; and 4, a ring of inflammatory cells more than four cells deep (maximum score = 8). The histological score for PBS/PBS control mouse lung tissue was always 0. At least six separate areas from similar sections within a single mouse were assessed; moreover, at least five mice were assessed. The five best sections were used for evaluation. PMNs were counted by an examiner who was blinded to the experimental group and are expressed as the number of cells per high power field.

**Statistical analyses**

For in vitro study, at least four independent experiments were performed with cultured cells from each donor, and the results are presented as the mean value ± standard deviation (SD) of four independent cultures. Differences between treatment groups were evaluated by analysis of variance (ANOVA) with a *post hoc* test. We present the *in vivo* results of real-time PCR, plaque assays, and ELISA as mean values ± SD from five individual mice and statistical significance of values between two compared groups was determined by Mann-Whitney tests. Statistical analyses were performed with GraphPad Prism software (version 5; GraphPad Software, La Jolla, CA, USA). A *p*-value <0.05 was considered to be statistically significant.

Supplementary figure S1

Transcripts encoding of TLR3, RIG-I, and MDA5 in NHNE cells treated with *S. epidermidis* and IAV relative to those in cells infected with IAV alone


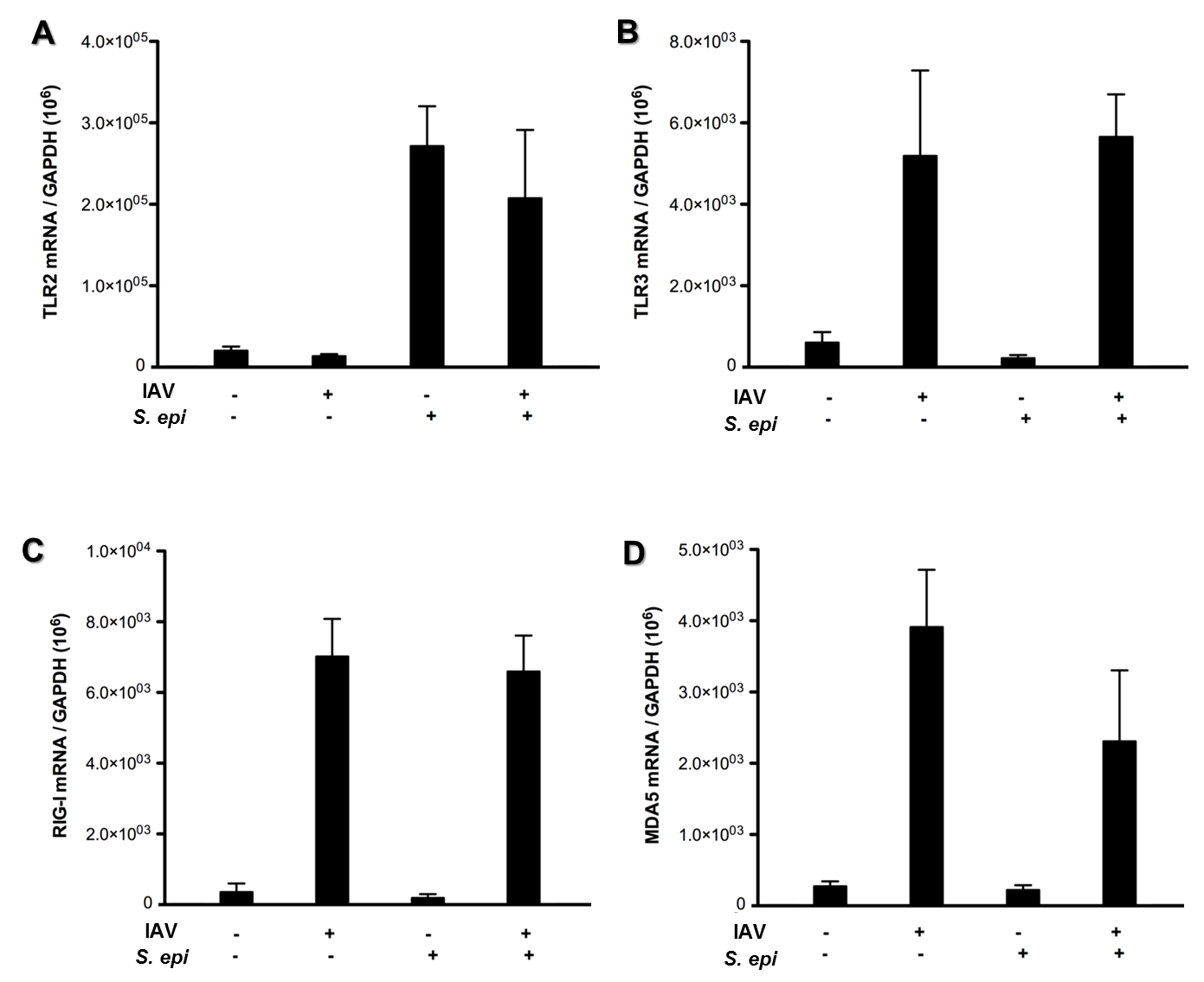


Supplementary figure S2. Microscopic finding of *S. epidermidis* colony in LB plate


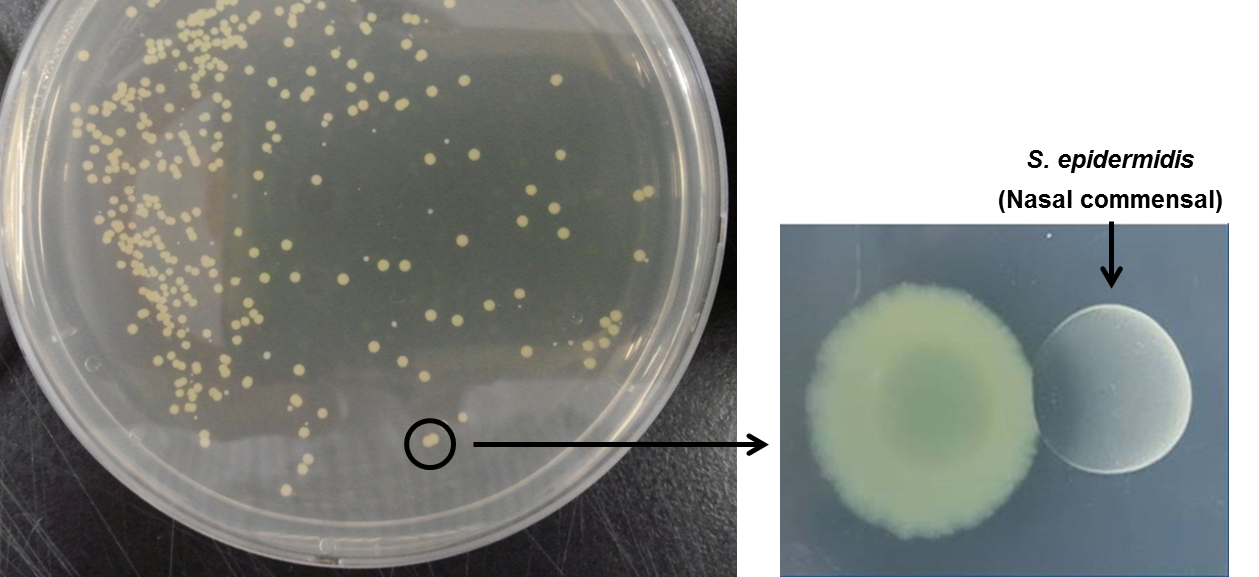

Supplement: Supplementary file 1 — Supplementary material and methods with supplementary figures. (DOC 1276 kb) [file 40168_2019_691_MOESM1_ESM.doc]
